# C(− 106)T polymorphism in ALR2 and risk of microvascular complications in T2DM patients in north Indian population

**DOI:** 10.1186/s43556-022-00087-y

**Published:** 2022-08-03

**Authors:** Archana Mishra, Mohammad Kaleem Ahmad, Haseeb Ahsan, Saba Khan, Sudhir Mehrotra, Roshan Alam

**Affiliations:** 1 Department of Biochemistry, Integral Institute of Medical Sciences and Research (IIMS&R), Integral University, Lucknow, 226026 India; 2 Present Address: Department of Biochemistry, Motilal Nehru Medical College, Prayagraj, 211002 India; 3 Department of Biochemistry, King George’s Medical University (KGMU), Lucknow, 226003 India; 4 Department of Biochemistry, Faculty of Dentistry, Jamia Millia Islamia, New Delhi, 110025 India; 5 Department of Medicine, TS Misra Medical College and Hospital, Amausi, Lucknow, 226008 India

Dear Editor,

A number of factors influence the prevalence and incidence of diabetes mellitus (DM), therefore, it is critical to identify these factors in order to prevent such disorders. Some patients with a short history of diabetes suffer microvascular complications, although they have relatively good glycemic control. Some patients, on the other hand, do not develop microvascular complications even with long-term disease and poor glycemic control, which could be attributed to genetic factors. Furthermore, there is familial aggregation for the occurrence of vascular problems in diabetes, suggesting the role of genetic predisposition in patients [1]. There is strong evidence that genes are one of the key contributors to diabetic microvascular complications besides environmental factors. A number of genes are involved in the etiology of microvascular complications with several polymorphic forms responsible for the development and progression of type 2 diabetes mellitus (T2DM). The single nucleotide polymorphisms (SNPs) of genes associated with diabetic microvascular complications were found to have a significant impact on the pathology of DM [2].

Several biochemical factors linked to DM include advanced glycation end products (AGEs), aldose reductase (AR), protein kinase C (PKC) acting through diacylglycerol (DAG) contributes to diabetic complications [3]. AR is the rate-limiting enzyme of polyol pathway which catalyzes the reduction of glucose to sorbitol utilizing the cofactor NADPH. AR plays an important role in DM through increase in sorbitol and reduction in myo-inositol due to upregulation of gene and increased enzyme activity. The polymorphisms in AR gene (ALR2) could be a key factor that regulates genetic susceptibility to diabetic complications [4, 5]. In ALR2, a change at position 106 of the promoter region, in which a thymine (T) is replaced with a cytosine (C) residue, leads to the formation of polymorphic form of the gene (C-106 T) in DM resulting in modulation of homeostasis, particularly depletion of NADPH and accumulation of sorbitol in cells/tissues leading to altered NADH/NAD ratio. The membrane leakage of glutathione (GSH) and myo-inositol results in hyperosmotic swelling leading to oxidative damage. The variation in the genetic sequence of ALR2 locus may cause overexpression of the gene, increasing the risk of DM. The enzyme activity may be genetically modulated due to polymorphism in the promoter or coding region of ALR2 [5, 6].

In the present study, we determined the association of C(− 106)T polymorphism in ALR2 and its relevance to T2DM microvascular complications in the North Indian diabetic population. All patients were subjected to a through clinical examination for the diagnosis of diabetic neuropathy, microalbuminuria and renal function tests were done to detect diabetic nephropathy and fundus examination for the detection of diabetic retinopathy (Supplementary material: Study subjects and patient selection). The BMI were significantly (*p* < 0.001) increased in T2DM and T2DM with microvascular complication patients as compared to healthy controls. We observed a significant increase in glycated hemoglobin (HbA1c) in T2DM patients and T2DM patients with microvascular complications compared to control group (*p* < 0.0001). The HbA1c of healthy control, T2DM patients and T2DM patients with microvascular complication were 5.13 ± 0.36%, 6.45 ± 1.07% and 7.75 ± 1.45%, respectively. The fasting blood sugar (FBS) in healthy control, T2DM patients and T2DM patients with microvascular complications were 90.98 ± 10.04 mg/dl, 153.13 ± 33.54 mg/dl and 184.82 ± 31.33 mg/dl, respectively (*p* < 0.001). The post-prandial blood sugar (PPBS) in healthy controls, T2DM patients and T2DM patients with microvascular complication were 116.37 ± 8.91 mg/dl, 212.75 ± 52.47 mg/dl and 256.64 ± 52.83 mg/dl, respectively (*p* < 0.001) (Supplementary Table 1).

The genomic DNA samples were amplified through the polymerase chain reaction (PCR) as shown in Supplementary Table 2*.* The PCR-RFLP analysis of products showed 206 bp and 57 bp fragments of homozygous CC; 147 bp, 59 bp and 57 bp for homozygous TT and 206 bp, 147 bp, 59 bp, 57 bp for heterozygous CT genotypes (Supplementary Fig. 1). Table 1 shows the C(− 106)T genotype and allele distribution of healthy controls, T2DM and T2DM patients with microvascular complications. The odd ratio of heterozygous CT and homozygous TT genotype (OR: 1.04, 95% CI: 0.583-1.875; *p* = 0.88) and (OR: 1.01% CI: 0.309-3.347; *p* = 0.97) found no significant association, including the T allele (OR: 1.03; 95% CI: 0.652-1.618; *p* = 0.90). The heterozygous CT genotype had an odd ratio of 1.27 (95% CI: 0.701-2.311; *p* = 0.52) and the homozygous TT genotype had the odd ratio of 3.304 (95% CI: 1.197-9.125; *p* = 0.03). The TT genotype was more common in T2DM with microvascular complication patients than normal controls. The T allele showed an odd ratio of 1.659 (95% CI: 1.075-2.561; *p* = 0.028) in T2DM with microvascular complications than normal controls. The homozygous TT genotype had the odd ratio of 3.25 (95% CI: 1.175-8.970; *p* = 0.034) with the T allele having an odd ratio of 1.615 (95% CI: 1.048-2.489; *p* = 0.038). Both the TT genotype and T allele were found to be significantly higher in T2DM with microvascular complications when compared to T2DM subjects.

**Table 1:** Comparison of genotypes and allele frequency of ALR2 between normal control, T2DM and T2DM with microvascular complication subjects

Genotype/Allele	Normal control (*N* = 100)	T2DM (N = 100)	T2DM with microvascular complications (*N* = 100)	OR (95%CI), *p*-value					
				Normal control vs T2DM		Normal control vs T2DM with microvascular complications		T2DM vs T2DM with microvascular complications	
CC	57 (57%)	56 (56%)	46 (46%)	Reference	–	Reference	–	Reference	–
CT	37 (37%)	38 (38%)	38 (38%)	1.04 (0.583-1.875)	0.88	1.27 (0.701-2.311)	0.52	1.217 (0.671-2.208)	0.61
TT	6 (6%)	6 (6%)	16 (16%)	1.01 (0.309-3.347)	0.97	3.304 (1.197-9.125)	0.03*	3.25 (1.175-8.970)	0.034*
C	151 (75.5%)	150 (75%)	130 (65%)	Reference	–	Reference	–	Reference	–
T	49 (24.5%)	50 (25%)	70 (35%)	1.03 (0.652-1.618)	0.90	1.659 (1.075-2.561)	0.028*	1.615 (1.048-2.489)	0.038*

Data are represented as counts. Percentages are shown in parenthesis (Wild type: CC genotype, Homozygous: TT Genotype, Heterozygous: CT Genotype)

* *p* < 0.05

Few studies have investigated the association of ALR2 polymorphism in diabetic microvascular complications in relation to the C-106 T locus (Supplementary material: Meta-analysis and comparison of results). Thus, the present study was undertaken to analyze the C(− 106)T polymorphism in ALR2 and its association with diabetic microvascular complications. We found that TT genotype (*p* = 0.034) was more common in T2DM with microvascular complications than T2DM patients. Both TT genotype and T allele were found to be more common in T2DM with microvascular complication than T2DM patients. In conclusion, our study demonstrated the significant association of C-106 T gene polymorphism in ALR2 (homozygous TT genotype) leading to microvascular complications in T2DM. Thus, genetic polymorphism/variability in ALR2 may be a useful for the diagnosis and prognosis of microvascular complications in T2DM.

To validate the role played by ALR2 polymorphism in various diabetic complications, further studies are required with a larger sample size and homogeneous genotyping methods. The ability of PCR-RFLP to detect nucleotide variations is limited by restriction enzymes/endonucleases (RE) to recognize some sequences. The real time-quantitative PCR (qPCR) method which detects SNPs should be applied for allele-specific PCR with high analytical sensitivity and specificity.

## Supplementary Information

**Additional file 1: Supplementary material.** **Supplementary Table 1.** Demographic/anthropometric profile of subjects/patients.** Supplementary Table 2.** Primer sequence and PCR amplification of ALR2. **Supplementary Figure 1.** BfaI polymorphism of ALR2 detected by PCR-RFLP. (Lane 1 ladder 100 bp, lane 2, 5, 7, 8 are CC Genotype and lane 3, 6 are CT Genotype lane 4, 9 are TT Genotype).

## Data Availability

The data generated or analyzed during the study is included in the letter. Data may also be available from the corresponding author upon reasonable request.

## Abbreviations

T2DM: Type 2 diabetes mellitus
Wild type: CC genotype
Homozygous: TT Genotype
Heterozygous: CT Genotype
BMI: Body mass index
SNPs: Single nucleotide polymorphisms
ALR2: Aldose reductase gene
HbA1c: glycated hemoglobulin
AGEs: Advanced glycation end products
AR: Aldose reductase enzyme
PKC: Protein kinase C
DAG: Diacylglycerol
FBS: Fasting blood sugar
PPBS: Post-prandial blood sugar
PCR: Polymerase chain reaction
RFLP: Restriction fragment length polymorphism
