# Supplementary material for: C(− 106)T polymorphism in ALR2 and risk of microvascular complications in T2DM patients in north Indian population
Source: Mol Biomed. 2022 Aug 3;3:24. doi: 10.1186/s43556-022-00087-y (PMC9346037; doi:10.1186/s43556-022-00087-y)
Supplement: Supplementary file 1 — Additional file 1: Supplementary material. Supplementary Table 1. Demographic/anthropometric profile of subjects/patients. Supplementary Table 2. Primer sequence and PCR amplification of ALR2. Supplementary Figure 1. BfaI polymorphism of ALR2 detected by PCR-RFLP. (Lane 1 ladder 100 bp, lane 2, 5, 7, 8 are CC Genotype and lane 3, 6 are CT Genotype lane 4, 9 are TT Genotype). [file 43556_2022_87_MOESM1_ESM.docx]

**SUPPLEMENTARY MATERIAL**

**Material and Methods**

1. ***Study subjects and patient selection***

The study was carried out after taking informed consent from patients and approval of the Institutional Ethical Committee (IEC), Integral Institute of Medical Sciences and Research (IIMS&R), Lucknow, India (IEC approval no. IEC/IIMS&R/2017/11-1). A total of 300 subjects were included which were divided into 100 patients of T2DM without microvascular complication, 100 patients of T2DM with microvascular complication and 100 healthy controls. All patients were subjected to a through clinical examination including detailed history for the diagnosis of diabetic neuropathy, microalbuminuria and renal function tests were done to detect diabetic nephropathy and fundus examination for the detection of diabetic retinopathy [1-3]. The T2DM group without microvascular complication comprised of 51 males and 49 females. The T2DM group with microvascular complications consisted of 53 males and 47 females. The study also recruited healthy controls in which 56 were males and 44 females. The mean age and body mass index (BMI) of healthy controls, T2DM and T2DM with microvascular patients were 53.46±7.01, 55.18±7.45, 57.18±5.31 years and 23.14± 3.45 kg/m^2^, 26.38 ±4.07kg/m^2^ and 27.58±3.87 kg/m^2^, respectively *(Supplementary Table 1).*

1. ***Isolation of genomic DNA and PCR***

The genomic DNA was extracted from whole blood cells using a QIAamp DNA blood Mini Kit (Qiagen, Chatsworth, CA, USA) and the purified DNA was loaded onto a 1% agarose gel containing ethidium bromide. The quantity and purity of DNA were determined by Nano Drops 1000 Spectrophotometer and the samples were stored at -40^o^C. The genomic DNA samples were amplified by polymerase chain reaction (PCR). The primers for PCR amplification and conditions used for thermal cycler are given in *Supplementary Table 2.* A 263 bp genomic DNA fragment of ALR2 was amplified and its size was verified on a 2% agarose gel electrophoresis. The PCR products were digested with restriction endonucleases Bfa1 overnight at 37^0^C. Then 10 μl of the products were loaded onto a 3.5% agarose gel. The restriction analysis of PCR products showed 206 bp and 57 bp fragments of homozygous CC; 147 bp, 59 bp and 57 bp for homozygous TT and 206 bp, 147 bp, 59 bp, 57 bp for heterozygous CT genotypes *(Supplementary Fig. 1).*

1. ***Statistical Analysis***

The present study was performed following the STROBE guidelines for reporting of genetic association studies. The sample size was calculated by using the formula:

where n = required sample size

r = ratio of controls to cases

Z_β_ = the desired power (0.84 for 80%)

Z_α/2_ = level of statistical significance (1.96)

P1 = proportion exposed in the case group

p2 = proportion exposed in control group

Thus, the sample size as calculated with the above formula is 98 in each case and control and the total no of sample is 294. Hence total of 300 subjects were included, 100 of T2DM patients without microvascular complications, 100 of T2DM patients with microvascular complication and 100 apparently healthy controls.

The statistical analysis was done using SPSS software version 23. The phenotypic data was compared using the ANOVA or unpaired t test. Values are given as mean SD (standard deviation). Allelic and genotypic frequencies were presented with 95% confidence interval (CI) and were analysed using χ^2^ test. Genotypes were tested for Hardy-Weinberg equilibrium. A p value <0.05 was considered statistically significant for the data analysed.

**Results**

***Meta-analysis and comparison of results***

Genotyping of C-106T polymorphism was performed using the polymerase chain reaction (PCR)-restriction fragment length polymorphism (RFLP) method described by Kao et al. [4]. The distributions of genotypes of the C-106T polymorphism did not differ from Hardy-Weinberg expectation for cases (chi-square 2.79; P 0.095) and controls (chi-square 1.79; P 0.18) [4]. Kao et al., [4] identified a novel polymorphism in the basal promoter region: a single substitution C(-106)T. For homozygote CC subjects, the 263-bp polymerase chain reaction (PCR) product contained only a single BfaI site, at which the product was cleaved into 206- and 57-bp fragments. The C(-106)T substitution created another BfaI restriction site. For homozygote TT subjects, the 206-bp fragment was further cleaved into 147- and 59-bp fragments. Heterozygotes (CT genotypes) therefore showed 206-, 147-, 59-, and 57-bp fragments. The frequency of the T allele was 35% in non-diabetic control subjects. Makiishi *et. al.*, [5] in a study on genetic polymorphism on diabetic nephropathy found that the TT genotype of the C-106T polymorphism of Aldo-Keto Reductase Family 1 Member B (AKR1B1) increases the risk for DN in Japanese subjects with T2DM which was similar to our study. Abu-Hassan et.al., [6] in a study on ALR2 gene polymorphism in Jordian T2DM patients demonstrated that the T allele was associated with increased risk of developing retinopathy in diabetic patients. Sivenius *et. al* [7], found that C(-106)T polymorphism in AKR1B1 gene was responsible for high risk of microalbuminuria which was similar to our study. In a study conducted on Iranian population it was found that diabetic patients carrying the T allele of C(‑106)T polymorphism in the ALR2 gene had a significantly higher urinary albumin excretion rate and microalbuminuria than subjects with C(‑106)C genotype and the T allele of the C(‑106)T polymorphism of the ALR2 gene in T2DM patients was associated with diabetic neuropathy. But in contrast, they found that diabetic patients with the T allele of the ALR2 gene was linked with lower rate of diabetic retinopathy than subjects with the C(‑106)C genotype, which could be due to ethnic differences [8]. In a study, Marzouk *et.al.,* [9] found a high frequency of CC genotype among diabetic patients with microvascular (58.7%) or without microvascular (53.3%) complications with no statistical significance. The CT genotypes (28.3%) in diabetic patients with microvascular complications was 14 (46.7%) whereas TT genotypes was 12 (13.0%) among diabetic patients with microvascular complications while no TT genotype was found in diabetic patients without microvascular complications. In a study on Japanese T2DM patient, it was found that C allele at position-106 in the promoter region of ALR2 gene was a susceptible allele for diabetic retinopathy as opposed to our results [10].

## Supplementary Table 1. Demographic/anthropometric profile of subjects/patients

| S.No. | Parameters/Variables | Healthy Control | T2DM | T2DM with microvascular complications | p value |
| --- | --- | --- | --- | --- | --- |
| 1 | **Age (Years)** | 53.46±7.01 | 55.18±7.45 | 57.86±5.13 | 0.0001*** |
| 2 | **BMI (kg/m^2^)** | 23.14± 3.4 | 26.3±4.07 | 27.58±3.87 | 0.0001*** |
| 3 | **HbA1c (%)** | 5.13±0.36 | 6.45±1.07 | 7.75±1.45 | 0.0001*** |
| 4 | **FBS (mg/dl)** | 90.98±10.04 | 153.13±33.54 | 184.82±31.33 | 0.0001*** |
| 5 | **PPBS (mg/dl)** | 116.37±8.91 | 212.70±52.47 | 256.64±52.83 | 0.0001*** |

**Supplementary Table 2.** Primer sequence and PCR amplification of ALR2

| Gene | Primers | Cycler condition |  |  |
| --- | --- | --- | --- | --- |
| ALR2 | Forward  5’CCTTTTCTGCCACGCGGGGCGCGGC-3’  Reverse  5’CATGGCTGCTGCGCTCCCCAG-3’ | 35 cycles | Initial Denaturation  Denaturation  Annealing  Extension  Final elongation | 95^o^C - 2 min  95^o^C - 1 min  70^o^C - 35 S  72^o^C - 35 S  72^o^C - 7 min |

**References**

1. Mishra A , Ahmad MK , Mehrotra S , Alam R, Ahmad I, Thapa P, Tiwari V. A study to investigate the possible association between GSTM1 AND GSTT1 polymorphism and the occurrence of complications in type 2 DM. Biochem Cell Arch. 2019; 19(2): 4485-4490. DOI : 10.35124/bca.2019.19.2.4485.
2. Selvin E, Marinopoulos S, Berkenblit G, Rami T, Brancati FL, Powe NR, Golden SH. Meta-analysis: glycosylated hemoglobin and cardiovascular disease in diabetes mellitus. Ann Intern Med. 2004; 141(6): 421-31. DOI: 10.7326/0003-4819-141-6-200409210-00007.
3. Stratton IM, Adler AI, Neil HA, Matthews DR, Manley SE, Cull CA, Hadden D, Turner RC, Holman RR. Association of glycaemia with macrovascular and microvascular complications of type 2 diabetes (UKPDS 35): prospective observational study. BMJ. 2000; 321(7258): 405-12. DOI: 10.1136/bmj.321.7258.405.
4. Kao Y-L, Donaghue K, Chan A, Knight J, Silink M. A novel polymorphism in the aldose reductase gene promoter region is strongly associated with diabetic retinopathy in adolescents with type 1 diabetes. Diabetes. 1999; 48: 1338-1340. DOI:10.2337/diabetes.48.6.1338.
5. Makiishi T, Araki S, Koya D, Maeda S, Kashiwagi A, Haneda M. C-106T polymorphism of AKR1B1 is associated with diabetic nephropathy and erythrocyte aldose reductase content in Japanese subjects with type 2 diabetes mellitus. Am J Kidney Dis. 2003; 42(5): 943-51. DOI: 10.1016/j.ajkd.2003.06.003

6. Abu-Hassan DW, Al-Bdour MD, Saleh I, Freihat M, El-Khateeb M. The relationship between aldose reductase gene C106T polymorphism and the severity of retinopathy in Type 2 diabetic patients: A case-control study. J Res Med Sci. 2021; 26:2. DOI: 10.4103/jrms.JRMS_250_20

7. Sivenius K, Niskanen L, Voutilainen-Kaunisto R, Laakso M, Uusitupa M. Aldose reductase gene polymorphisms and susceptibility to microvascular complications in Type 2 diabetes. Diabet Med. 2004; 12: 1325-33. DOI: 10.1111/j.1464-5491.2004.01345.x.

8. Rezaee MR, Amiri AA, Hashemi-Soteh MB, Daneshvar F, Emady-Jamaly R, Jafari R, Soleimani B, Haghiaminjan H. Aldose reductase C‑106T gene polymorphism in type 2 diabetics with microangiopathy in Iranian individuals. Ind J Endocrinol Metabol. 2015; 19(1): 95-99. DOI: 10.4103/2230-8210.131762.

9. Marzouk SA, Abla A Abou Zied, Nermine H Zakaria and Eman S Gharraf. Role of aldose reductase C-106T polymorphism among diabetic Egyptian patients with different microvascular complications. Am J Exp Clin Res. 2014; 1(2): 18-24. DOI: NA

10. Katakami N, Kaneto H, Takahara M. et al. Aldose reductase C-106T gene polymorphism is associated with diabetic retinopathy in Japanese patients with type 2 diabetes. Diab Res Clin Pract. 2011; 15: 8-9. DOI: 10.1016/j.diabres.2011.02.017.
